# Responsive Thalamic Neurostimulation: A Systematic Review of a Promising Approach for Refractory Epilepsy

**DOI:** 10.3389/fnhum.2022.910345

**Published:** 2022-07-05

**Authors:** Chaim M. Feigen, Emad N. Eskandar

**Affiliations:** Department of Neurological Surgery, Montefiore Medical Center, Bronx, NY, United States

**Keywords:** thalamic nuclei, epilepsy, deep brain stimulation, responsive neurostimulation system, neuromodulation

## Abstract

**Introduction:**

Responsive neurostimulation is an evolving therapeutic option for patients with treatment-refractory epilepsy. Open-loop, continuous stimulation of the anterior thalamic nuclei is the only approved modality, yet chronic stimulation rarely induces complete seizure remission and is associated with neuropsychiatric adverse effects. Accounts of off-label responsive stimulation in thalamic nuclei describe significant improvements in patients who have failed multiple drug regimens, vagal nerve stimulation, and other invasive measures. This systematic review surveys the currently available data supporting the use of responsive thalamic neurostimulation in primary and secondary generalized, treatment-refractory epilepsy.

**Materials and Methods:**

A systematic review was performed using the following combination of keywords and controlled vocabulary: (“Seizures”[Mesh] AND “Thalamus”[Mesh] AND “Deep Brain Stimulation”[Mesh]) OR (responsive neurostim* AND (thalamus[MeSH])) OR [responsive neurostimulation AND thalamus AND (epilepsy OR seizures)]. In addition, a search of the publications listed under the PubMed “cited by” tab was performed for all publications that passed title/abstract screening in addition to manually searching their reference lists.

**Results:**

Ten publications were identified describing a total of 29 subjects with a broad range of epilepsy disorders treated with closed-loop thalamic neurostimulation. The median age of subjects was 31 years old (range 10–65 years). Of the 29 subjects, 15 were stimulated in the anterior, 11 in the centromedian, and 3 in the pulvinar nuclei. Excluding 5 subjects who were treated for 1 month or less, median time on stimulation was 19 months (range 2.4–54 months). Of these subjects, 17/24 experienced greater than or equal to 50%, 11/24 least 75%, and 9/24 at least 90% reduction in seizures. Although a minority of patients did not exhibit significant clinical improvement by follow-up, there was a general trend of increasing treatment efficacy with longer periods on closed-loop thalamic stimulation.

**Conclusion:**

The data supporting off-label closed-loop thalamic stimulation for refractory epilepsy is limited to 29 adult and pediatric patients, many of whom experienced significant improvement in seizure duration and frequency. This encouraging progress must be verified in larger studies.

## Introduction

Epilepsy, defined as recurring unprovoked seizures, (Blume et al., 2001) is a chronic, debilitating neurologic condition affecting up to 1% of the global population (Liu et al., 2013; Perez-Malagon and Lopez-Gonzalez, 2021). While antiepileptic drugs (AED) remain the gold standard of treatment, (Zhong et al., 2011; Yu et al., 2018) the World Health Organization estimates one-third of patients do not respond adequately to AEDs; these individuals are considered to have treatment refractory epilepsy (TRE) (Kwan and Brodie, 2000; Liu et al., 2013; Perez-Malagon and Lopez-Gonzalez, 2021; Velasco et al., 2021). The mortality rate for TRE is slightly greater than 1/1,000 person years, (Perez-Malagon and Lopez-Gonzalez, 2021) and advances in neuropharmacology over the last four decades have not significantly improved rates of seizure remission amongst the TRE population (Chen et al., 2018; Sisterson et al., 2019). While epilepsy surgery is a viable option for patients with an identified epileptogenic focus, (Yu et al., 2018) many individuals are not candidates for resection either because the seizure focus is located in eloquent cortex [motor or speech areas (Burdette et al., 2021)], there are multiple foci, or the seizures generalize without an identifiable focus (Morrell and RNS System in Epilepsy Study Group, 2011; Zhong et al., 2011). Additionally, some patients who are appropriate candidates for surgery may be hesitant to commit to irreversible ablation or resection (Thomas and Jobst, 2015; Perez-Malagon and Lopez-Gonzalez, 2021).

Approved in 1995, vagal nerve stimulators (VNS) were the first implantable neuromodulation devices for epilepsy (Ryvlin and Jehi, 2022). While there are some reports of success, most patients with TRE who receive VNS do not achieve adequate seizure relief (Zhong et al., 2011). Notwithstanding these limitations, the significant improvement seen in some patients implanted with VNS highlights the potential for using electrical stimulation of neural tissue to prevent or abort seizures (Boon et al., 2009; Zhong et al., 2011). There is a pressing need to develop additional treatment modalities for children and adults with TRE who are not candidates for epilepsy surgery (Zhong et al., 2011). The efforts by clinicians and physiologists to develop alternative treatment modalities for these vulnerable patients has brought renewed interest in exploring the effects of stimulating subcortical structures contained in epileptic circuits (Goldstein and Harden, 2000; Kerrigan et al., 2004).

The field of neurostimulation for TRE has been progressing over the last four decades with the first case of DBS targeting the anterior nucleus of the thalamus (ANT) reported by Cooper et al. (1980). Several years thereafter, Fisher et al. (1992) implanted programmable stimulators into the bilateral centromedian nucleus of the thalamus (CMT) of seven patients with intractable epilepsy, demonstrating the feasibility of controlled trials for thalamic stimulation. In a follow-up landmark study, Fisher conducted the Stimulation of the ANT for Epilepsy (SANTE) trial, in which 110 patients with TRE were randomly assigned to 3 months of either DBS or sham treatment through bilateral ANT implants followed by unblinding and an additional DBS phase. The SANTE authors reported greater than 50% seizure frequency reduction in at least half of the participants at 2 years follow-up, with no serious complications including death, hemorrhage or infections arising from the DBS implants (Fisher et al., 2010). A minority of subjects experienced adverse effects including depression, memory loss and cognitive impairment during the initial, blinded phase of the study. Neurocognitive assessments conducted after the completion of the trial showed that these adverse effects resolved, at the latest, within several months post-stimulation (Fisher et al., 2010; Hartikainen et al., 2014). The SANTE subjects were prospectively followed for an additional 5 years with a reported 69% reduction in seizures. The results of the trial demonstrated the efficacy and safety of thalamic DBS in treating patients with TRE (Salanova et al., 2015). Subsequent studies supported the use of DBS in focal regional epilepsy by flanking the epileptic region with two leads or by combining neocortical and thalamic stimulation (Burdette et al., 2021). The results of the trial led to FDA approval of ANT DBS in 2018- the only deep brain structure for which stimulation is an approved treatment for TRE (Rincon et al., 2021).

The RNS^®^ System^1^ (NeuroPace, Mountain View, CA, United States) is a closed-loop device that features two 4-contact electrode leads connected to a neurostimulator. One or two leads are implanted at seizure foci to detect epileptiform activity and deliver stimulation aimed at aborting incipient seizures. The detection system monitors (1) EEG signal intensity, (2) changes in wave frequency, and (3) discharges within pre-specified frequency ranges in order to identify epileptiform activity (Thomas and Jobst, 2015). The system responds to evolving seizures with stimulation to block or terminate the epileptic activity (Wong et al., 2019; Burdette et al., 2020). The device is most commonly employed in bilateral temporal epilepsy, left temporal lobe epilepsy where the risk for memory following ablation or resection is too great, or for seizures arising in or adjacent to eloquent cortex (Kokoszka et al., 2018). The pulse generator is implanted in a recess in the skull, and has a mean battery life of 3–4 years (Wong et al., 2019). Controlled trials utilizing responsive stimulation in the neocortex have demonstrated that both open- as well as closed-loop modalities afford patients significant seizure reduction compared to sham stimulation, with similar rates of implant complications (Heck et al., 2014; Bergey et al., 2015). Patients implanted with the system upload their intracranial electroencephalography (EEG) data to an online repository. The RNS^®^ system gained FDA approval in 2013 for adults (≥18 years) with partial-onset seizures in one or two foci which fail to significantly improve on ≥2 AEDs (Kokoszka et al., 2018; Kwon et al., 2020). An overview of RNS detection and stimulation parameters has been previously published (Sisterson et al., 2019).

Open-loop continuous stimulation is the only thalamic stimulation modality approved for epilepsy, yet chronic thalamic stimulation rarely induces complete seizure remission, and is associated with neuropsychiatric adverse effects (Ryvlin et al., 2021). Studies of open-loop thalamic stimulation have been previously reviewed, the results of which highlight the safety and efficacy of thalamic stimulation as an alternative modality to permanent surgery (Zhong et al., 2011; Zangiabadi et al., 2019). Similarly, accounts of off-label responsive stimulation in a variety of thalamic nuclei report significant improvement in seizure control for patients who have failed drug therapy, vagal nerve stimulation and other invasive measures (Osorio et al., 2005; Kokoszka et al., 2018; Elder et al., 2019; Herlopian et al., 2019; Burdette et al., 2020, 2021; Kokkinos et al., 2020; Kwon et al., 2020; Welch et al., 2021). These reports highlight the advantages of having the ability to continuously record and temporarily interrupt major neural networks involved in the propagation of idiopathic generalized seizures. No review to date has consolidated the literature describing results from patients with TRE who underwent closed-loop thalamic neurostimulation. Accordingly, the purpose of this systematic review is to provide a synopsis of primary research publications through May 2022 describing clinical outcomes of closed-loop responsive thalamic stimulation in patients with TRE.

## Materials and Methods

The review was performed according to the guidelines of the Preferred Reporting Items for Systematic Reviews and Meta-Analysis Statement (PRISMA) (Shamseer et al., 2015). Eligibility criteria included case reports, case series and retrospective analyses of patients with TRE who were treated with closed loop responsive thalamic neurostimulation. Exclusion criteria included (1) review papers, (2) studies describing open-loop thalamic stimulation for epilepsy, (3) animal studies, (4) studies that did not describe treatment effects following responsive thalamic stimulation (“outside the scope”), (5) letters to editors, (6) studies describing responsive thalamic stimulation but do not present follow-up data, and (7) meeting reports. In addition, if studies describe multiple patients but only provided follow-up data for a subset of patients, then the publication would be included in the review, but only the subset of patients for whom follow up data were available would be discussed in the results.

An examination of publications was carried out by searching PubMed from inception through May 2022 for articles published in peer-reviewed journals. The search strategy utilized a combination of keywords and controlled vocabulary terms (MeSH). Search results were restricted to English language only. Search terms included (“Seizures”[Mesh] AND “Thalamus”[Mesh] AND “Deep Brain Stimulation”[Mesh]) OR (responsive neurostim* AND (thalamus[MeSH])) OR [responsive neurostimulation AND thalamus AND (epilepsy OR seizures)]. In addition, a search of the publications listed under the PubMed “cited by” tab was performed for all included references from the initial search reports, and manual searches of the reference sections of the included studies were performed as well. All references were exported into Microsoft Excel for duplicate removal, screening, and data extraction.

## Results

### Literature Search

**FIGURE 1:**
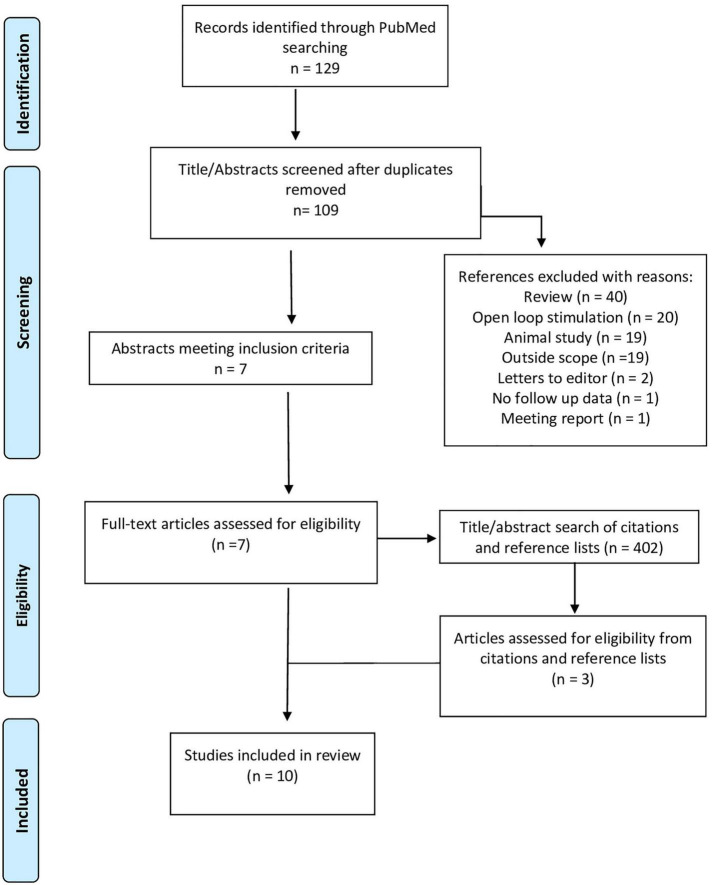
PRISMA flow diagram.

After removal of duplicates, 109 references underwent title and abstract screening resulting in the identification of 7 publications which met the inclusion criteria (Osorio et al., 2005; Herlopian et al., 2019; Burdette et al., 2020, 2021; Kokkinos et al., 2020; Welch et al., 2021; Beaudreault et al., 2022). A supplemental search of the reference lists and articles which cited these 7 publications was performed as well, yielding 3 additional publications (Kokoszka et al., 2018; Elder et al., 2019; Kwon et al., 2020; Figure 1, PRISMA Diagram). The identified publications describe a total of 29 subjects with a broad range of epilepsy subtypes who were treated with closed-loop thalamic neurostimulation. The median age of subjects was 19 years old (range 10–65 years). All subjects had failed to control their seizures with ≥3 AEDs, 10 subjects (34%) failed to improve with VNS treatment, and 11 (38%) had previously undergone surgical ablation or resection without seizure remission. At most recent follow up, 15 subjects were receiving stimulation in the ANT (7 unilateral and 8 bilateral nuclei), 11 in the CMT (2 unilateral and 9 bilateral) and three subjects in the PVN (2 unilateral and 1 bilateral). In addition, Beaudreault et al. (2022) included two subjects in their 14-patient case series for whom follow-up data was not available; the other 12 subjects were included in this analysis.

While most of the studies described the effects of responsive thalamic stimulation after a treatment period of 6 months or greater, Osorio et al. (2005) reported 60% seizure reduction in four patients after a median 6.3 days of treatment and Kokoszka et al. (2018) reported 50% reduction in one patient after 1 month of treatment. The median time on responsive thalamic stimulation for the other subjects was 19 months (range 2.4–54 months). Of the subjects who received responsive thalamic stimulation for ≥6 months, 17/24 subjects experienced greater than or equal to 50% reduction, 11/24 at least 75% percent reduction, and 9/24 achieved 90% reduction in daily seizure frequency. A summary of subjects’ histories of epilepsy, treatment regimens, and outcomes appears in Table 1.

**TABLE 1:** Subjects treated with thalamic responsive neurostimulation for refractory epilepsy.

Age (Y)	Sex	Disabling seizure type	Previous surgery	Location stimulating leads	Location detection leads	Time on stimulation	% reduction seizures^†^	Year	Author	Journal
31	N/A	Mesial temporal partial with ± secondary generalization	None	Bilateral ANT	amigdalohippocampal area	10.2D	72.9 (all seizures)	2005	Osorio	Annals of Neurology
31	N/A	Mesial temporal partial with ± secondary generalization	None	Bilateral ANT	amigdalohippocampal area	2.8D	75.6 (all seizures)	2005	Osorio	Annals of Neurology
31	N/A	Mesial temporal partial with ± secondary generalization	None	Bilateral ANT	amigdalohippocampal area	5.9D	42.8 (all seizures)	2005	Osorio	Annals of Neurology
31	N/A	Mesial temporal partial with ± secondary generalization	None	Bilateral ANT	amigdalohippocampal area	6.6D	48.8 (all seizures)	2005	Osorio	Annals of Neurology
14	M	FSG	frontal lobectomy; complete CC; VNS; TL; posterior QT	Unilateral ANT and right temporal strip	Right temporal strip	1M	50 (all seizures)	2018	Kokoszka	Journal of Neurosurgery Pediatrics
27	M	FSG and FIA	VNS; anterior TL; frontal parietal corticectomy; anterior CC	Unilateral ANT	Left ANT, left middle temporal gyrus	33M	50 (all seizures)	2019	Elder	Epilepsia
23	M	FSG and FIA	VNS	Unilateral ANT	Right ANT, right postcentral gyrus	33M	53.3 (all seizures)	2019	Elder	Epilepsia
24	M	FIA	None	Unilateral ANT	Left ANT, left parietal lobe	35M	56 (all seizures)	2019	Elder	Epilepsia
34	M	Genetic generalized epilepsy	Anterior CC; VNS	Unilateral ANT	Right lateral posterior frontal cortex	24M	>90 (all seizures)	2019	Herlopian	Annals of Clinical and Translational Neurology
11	F	Focal sensory and GTC	Focal resection	Right ANT left hippocampus	Right ANT left hippocampus	16M	75–99 (all seizures)	2022	Beaudreault	Frontiers in Human Neuroscience
14	M	LGS	Focal resection, CC, anterior commissurotomy, VPS, VNS	Bilateral ANT	Bilateral ANT	40.4M	0–24 (all seizures)	2022	Beaudreault	Frontiers in Human Neuroscience
14	N/A	Combined generalized and focal	None	Right ANT right anterior cingulate	Right ANT right anterior cingulate	46.8M	75–99 (all seizures)	2022	Beaudreault	Frontiers in Human Neuroscience
19	N/A	Localization related with secondary generalization	None	Bilateral ANT	Bilateral ANT	51.6M	0–24 (all seizures)	2022	Beaudreault	Frontiers in Human Neuroscience
10	N/A	LGS	CC	Bilateral ANT	Bilateral ANT	51.6M	25–49 (all seizures)	2022	Beaudreault	Frontiers in Human Neuroscience
17	N/A	Idiopathic generalized epilepsy	None	Bilateral ANT	Bilateral ANT	52.8M	75–99 (all seizures)	2022	Beaudreault	Front Hum Neurosci
22	N/A	Regional focal epilepsy	None	Unilateral CMT	CMT and epileptogenic cortical region	8M	55 (all seizures)	2020	Burdette	Epilepsy & Behavior
19	F	Eyelid myoclonia with absences	None	Bilateral CM/VL	Bilateral CM/VL	18M	84 (all seizures)	2020	Kokkinos	Neurosurgery
14	F	GTC secondary to LGS	Frontal cortical resection, VNS	Bilateral CMT	Bilateral CMT leads and bilateral frontopolar leads	20M	70–90 (drop attacks) 100 (GTC)	2020	Kwon	Annals of Clinical and Translational Neurology
12	M	GTC secondary to LGS	None	Bilateral CMT	Bilateral CMT leads and bilateral frontal cortical	12M	90 (drop attacks) 100 (GTC)	2020	Kwon	Annals of Clinical and Translational Neurology
16	M	Absence seizures with occasional secondary GTC	Stereotactic laser ablation of amygdala lesion	Bilateral CMT	Bilateral CMT	6M	66–75 (absence seizures)	2021	Welch	Frontiers in Neurology
29	N/A	Localization-related with impaired awareness and focal to GTC	Temporal lobectomy, VNS	Bilateral CMT	Bilateral CMT	54M	75–99 (all seizures)	2022	Beaudreault	Frontiers in Human Neuroscience
17	N/A	LGS	VNS	Bilateral CMT	Bilateral CMT	10.8M	25–49 (all seizures)	2022	Beaudreault	Frontiers in Human Neuroscience
16	N/A	LGS	None	Bilateral CMT	Bilateral CMT	8.4M	25–49 (all seizures)	2022	Beaudreault	Frontiers in Human Neuroscience
21	N/A	Generalized onset	None	Right CMT, left frontal	Right CMT, left frontal	27.6M	50–74 (all seizures)	2022	Beaudreault	Frontiers in Human Neuroscience
14	N/A	Idiopathic generalized epilepsy	VNS	Bilateral CMT	Bilateral CMT	2.4M	25–49 (all seizures)	2022	Beaudreault	Frontiers in Human Neuroscience
10	N/A	Localization-related with impaired consciousness	None	Bilateral CMT	Bilateral CMT	16.8M	0–24 (all seizures)	2022	Beaudreault	Frontiers in Human Neuroscience
31	N/A	Posterior quadrant focal epilepsy	Periventricular LITT previous cortical RNS	Bilateral PVN	Right PVN + right occipital, left PVN + left occipital	12.5M	>90 (disabling seizures)	2021	Burdette	Epilepsia
41	N/A	Posterior quadrant focal epilepsy	R ATL R STG resection	Unilateral PVN	Right PVN + right parietal	15M	60–70 (disabling seizures)	2021	Burdette	Epilepsia
65	N/A	Posterior quadrant focal epilepsy	None	Unilateral PVN	Right PVN + right occipitotemporal	10M	90 (disabling seizures)	2021	Burdette	Epilepsia

*^†^Type of seizure listed as specified by authors. ANT, anterior thalamic nucleus; CC, corpus callosotomy; CMT, centromedian thalamic nucleus; CM/VL, centromedian ventrolateral; FIA, focal with impaired awareness; FSG, focal with secondary generalization; GTC, generalized tonic clonic; LGS, Lennox Gastaut Syndrome; LITT, laster interstitial thermal therapy; PVN, pulvinar nucleus; QT, quadrantectomy; RNS, Responsive Neurostimulator; STG, superior temporal gyrus; TL, temporal lobectomy; VNS, vagal neurostimulator; VPL, ventriculoperitoneal shunt.*

### Responsive Stimulation in the Anterior Thalamic Nuclei: Acute Results

In the first report of closed loop thalamic stimulation to treat epilepsy, Osorio et al. (2015) used an externalized monitoring system for automated detection of seizure activity which triggered high frequency electrical stimulation (ES) in either cortical or thalamic targets (four subjects each). Eight subjects were initially assessed with depth electrodes in the bilateral hippocampus and strips placed over suspected cortical epileptic zones. Four subjects with ≥2 epileptogenic cortical foci were assigned to the “thalamic remote closed loop” cohort. This cohort had an average age of 31 years, and all subjects were diagnosed with mesial temporal partial epilepsy, with or without secondary generalization. Electrocorticography (ECoG) was performed using the same implants as in the control phase of the study and the patients were stimulated in the bilateral ANT using high frequency ES (100–500 MHz), which was triggered by seizure detection. The authors report a mean reduction of seizures per day from 1.36 in the control phase to 0.72 in the treatment phase, with a mean drop in seizure frequency of 55.5%. These results were the first empiric evidence that automated electrical stimulation of the thalamus during real time electrophysiologic monitoring is safe, tolerable, and efficacious.

Kokoszka et al. (2018) describe the first off-label use of the NeuroPace RNS © to target deep brain structures. The RNS system was implanted in a 14-year-old male with West syndrome and focal cortical dysplasia. Prior to VNS implant, the patient’s intractable focal seizures failed to improve with 4 AEDs, right frontal lobectomy, corpus callosotomy and VNS. Data from scalp recordings as well as grid and strip depth electrodes identified the left lateral frontal and right lateral temporal regions as the most highly epileptogenic foci and were selected as RNS strip electrode targets. Diagnostic left cortical stimulation using the strip electrodes increased seizure activity. The left strip electrode was removed, the right cortical strip was left for closed loop monitoring, and a depth electrode was activated in the left ANT. The patient experienced a 50% decrease in seizure frequency within 1 month and continued to improve with gradually increasing charge density. This was also the first reported use of thalamic RNS to treat epilepsy in a pediatric patient.

### Responsive Stimulation in the Anterior Thalamic Nuclei: Treatment for ≥6 Months

Elder et al. (2019) describe 3 adult, male patients, mean age 24.6 years, with longstanding multifocal epilepsy. All three subjects had focal seizures with impaired awareness and two had focal seizures with secondary generalization. All patients had failed multiple medications as well as some form of invasive treatment, including corpus callosotomy and/or VNS. The patients underwent ECoG monitoring revealing cortical epileptic zones. The RNS stimulation leads targeted the ANT as well as epileptogenic cortex. Two patients received bilateral ANT stimulation while the third received unilateral stimulation. All patients reported at least 50% reduction in seizures, with no adverse neurocognitive effects of RNS, at 33 months follow-up. This publication had the longest closed-loop corticothalamic stimulation period, with a mean duration up of 33.7 months. Additionally, this was the first study to demonstrate efficacy in closed-loop unilateral ANT stimulation.

**FIGURE 2:**
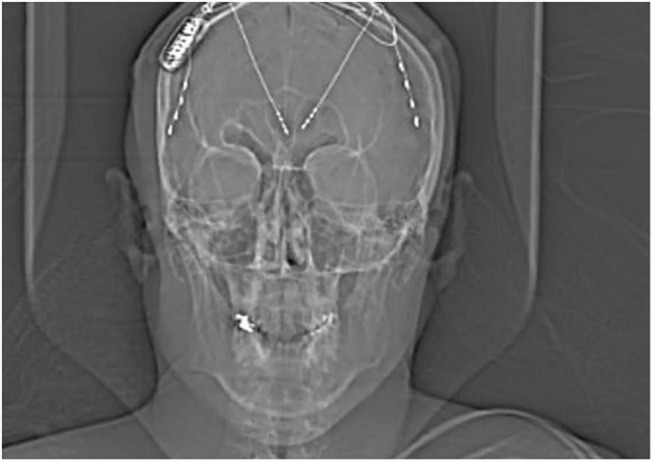
X-ray displaying the implantation of RNS device with 2-depth electrodes each with four contacts targeting the ANT and 2-strips in the prefrontal cortex of a 34-year-old patient with genetic generalized epilepsy; only the right ANT depth and right cortical strip were attached to the RNS device. Adapted with permission from Herlopian et al. (2019).

Herlopian et al. (2019) reported a 34-year-old male patient with intractable, childhood-onset, generalized epilepsy including tonic, atonic, and myoclonic absence seizures. The patient had failed multiple AEDs and underwent two rounds of invasive ECoG with anterior strip electrodes, anterior corpus callosotomy and VNS without significant improvement. The patient was re-evaluated for neurosurgery 13 years after callosotomy and had a non-revealing structural MRI, as well as positron emission tomography (PET) demonstrating antero-medial, bifrontal hypometabolism. To rule out cryptic frontal lobe epilepsy as the etiology of medication resistance, the patient underwent a third round of invasive monitoring with depth electrodes, which assisted in the determination of genetic generalized epilepsy with asymmetric thalamocortical propagation, most prominent in the lateral posterior frontal lobes. The seizures also had an overall left hemispheric predominance. The patient was treated with RNS targeting the right lateral posterior frontal cortex and right ANT (Figure 2). ECoG revealed that the targeted cortex and ipsilateral ANT were highly synchronized at the onset of ictal activity. Cortical detection with responsive stimulation at the thalamic lead was determined to be the most beneficial setting. The authors reported a 90–95% seizure reduction by 24 months and no adverse reactions to corticothalamic RNS. Finally, a large case series was recently published by Beaudreault et al. (2022) which included six subjects with active leads implanted in the ANT. The authors report successful seizure detection and stimulation using the same ANT leads.

In total, the 8/11 patients who received closed-loop stimulation targeting the ANT showed at least 50% improvement in seizure frequency after 1 month. These findings are consistent with the success seen in the pilot studies of open-loop, chronic DBS targeting the ANT (Hodaie et al., 2002; Kerrigan et al., 2004; Andrade et al., 2006; Lim et al., 2007; Osorio et al., 2007; Lee et al., 2012). The ANT remain the most widely targeted structure for treating epilepsy with neuromodulation (Zhong et al., 2011; Zangiabadi et al., 2019; Perez-Malagon and Lopez-Gonzalez, 2021).

### Responsive Stimulation in the Centromedian Thalamic Nuclei

Burdette et al. (2020) describe seven adult patients (mean age 33.4 years) with intractable focal epilepsy who received RNS with one lead in the CMT and a second lead in ipsilateral epileptogenic cortex, as identified by diagnostic stereo-EEG. All patients had failed to achieve remission with multiple AEDs and were not candidates for any form of epilepsy surgery. One patient demonstrated bilateral regional foci, and received 2 RNS systems, each with leads targeting CMT and ipsilateral cortex. At a median 17 months follow-up, the authors report a mean reduction in disabling epilepsy of 80% and a reduction of overall seizure activity of 67%.

Kokkinos et al. (2020) implanted RNS leads in the centromedian/ventrolateral (CM/VL) nuclei of a 19-year-old female with eyelid myoclonus plus absences who failed numerous AEDs. The patient received bilateral 4-contact RNS leads with the distal contacts at the CMT and most proximal contacts in the VL nuclei. One-year scalp EEG follow-up verified successful CM/VL stimulation by RNS during generalized spike-wave discharges. Within 18-months of closed-loop CM/VL stimulation, the patient had improved from an average 60 seizures/day to fewer than 10. This was the first report of successful thalamic RNS to treat absence seizures.

Kwon et al. (2020) targeted the CMT in two patients, aged 16 and 12, with Lennox-Gastaut Syndrome (LGS), and autism spectrum disorder. The 16-year-old female failed to improve with 17 different AEDs. Anterior cortical resection, and VNS treatment. She presented to the authors with frequent, complex, generalized seizures. Depth ECoG monitoring revealed multifocal, generalized seizure activity with diffuse regions of onset, predominantly in the right orbitofrontal cortex. The patient received an RNS system with bilateral CMT and frontopolar leads. Initially the RNS stimulator was connected to right frontopolar and left CMT leads, with 50% seizure improvement. It was later determined that cortical stimulation was not contributing to the therapeutic benefit of RNS, and the cortical lead was disconnected, with the lead moved to the right CMT. Bilateral CMT stimulation resulted in a 70–90% improvement in drop attacks and 100% improvement in both myoclonus and generalized tonic-clonic attacks by 26-month follow-up. The second patient was a 12-year-old male with LGS who had a 4-year history of frequent (up to 50/day) complex generalized seizures. The patient had failed 6 AEDs. Genetic testing was significant for Dup15q syndrome which is associated with autism and epilepsy. Structural MRI revealed callosal dysgenesis and colpocephaly without hydrocephalus. The authors implanted the RNS system with bilateral frontal cortical strips and bilateral CMT electrodes, with stimulation connected to both CMT leads. Within 12-month, the patient experienced a 75–95% seizure reduction. No adverse events were reported.

Welch et al. (2021) describe a 16-year-old male with classical 3 Hz spike-and-wave discharges which would intermittently accompany seizure activity. An amygdala lesion was detected on structural MRI, resembling a neuroepthelial tumor. Given the patient’s ambiguous radiologic findings and an evolving EEG, the patient underwent stereo EEG in the right temporal (including the lesion), right cingulate and left hippocampus. Invasive monitoring did not isolate an epileptic focus, but demonstrated widespread propagation of the spike-and-wave activity. The authors noted that the contacts immediately adjacent to the amygdala lesion detected intermittent 3 Hz spike-and-waves, but these were unrelated to clinical episodes and never evolved to generalized seizures. The lesion underwent stereotactic laser ablation, and biopsy revealed a low-grade, mixed glial and neuronal neoplasm. The patient received RNS with 4 contact depth electrodes targeting the bilateral CMT. RNS ECoG detected absence and GTC activity in the bilateral CMT. The authors report improvement within 1 month of starting RNS and complete cessation of absence seizures at 6 months. This case further highlights the efficacy of thalamic RNS in inducing remission of absence seizures. In addition, the recent review by Beaudreault et al. (2022) includes six subjects with a range of response rates to RNS treatment targeting the CMT.

### Pulvinar Nuclei

In the first case series reporting RNS in the thalamic pulvinar nuclei (PVN), Burdette et al. (2021) utilized the RNS system in three patients (mean age 45.7 years) diagnosed with multilobar posterior epilepsy. The first patient had previously undergone interstitial laser ablation for bilateral periventricular nodular heterotopia. This patient had two RNS systems implanted with 2 right sided occipital strips and 1 left sided occipital depth lead. ECoG demonstrated focal seizures arising independently in the left and right posterior quadrants. Stimulation was targeted at the bilateral PVN and occipital lobes. The remaining two patients received RNS implants in the right PVN and ipsilateral posterior quadrant. ECoG in the second patient demonstrated synchronized PVN activity with seizures arising in a variety of cortical locations and in both hemispheres. Evidence of PVN synchronization was weaker in the third patient, whose recordings showed epileptic activity arising predominantly in the right posterior quadrant, accompanied by voltage changes in the right PVN at seizure onset. The second patient had previously undergone right anterior temporal lobectomy and resection of remnant superior temporal gyrus, while the third patient did not have previous epilepsy surgery. The results of this study demonstrated that RNS in the PVN is a safe and efficacious modality for treating focal epilepsy originating in the posterior quadrants.

## Discussion

This review identified 10 publications where ECoG was coordinated with automated stimulation of thalamic nuclei triggered by epileptic discharges to treat refractory epilepsy. Osorio et al. (2005) describe an externalized system which combined electrophysiologic monitoring in the bilateral anterior temporal lobes with high-frequency stimulation of the ANT. The remaining 9 reports describe the off-label use of the RNS system to record from an assortment of corticothalamic locations and target a variety of thalamic nuclei (Kokoszka et al., 2018; Elder et al., 2019; Herlopian et al., 2019; Burdette et al., 2020, 2021; Kokkinos et al., 2020; Kwon et al., 2020; Welch et al., 2021). 24 out of the 29 subjects exhibited significant reduction in seizure frequency with responsive thalamic stimulation, after failing multiple AEDs.

Early observations of decerebrate animals produced evidence supporting both the “centrencephalic” and “telencephalic” theories of epilepsy which state that generalized epilepsy spreads throughout the cortex from nuclei in the brainstem, (Gastaut et al., 1969; Velasco et al., 2021) or from discreet cortical foci by crossing the commissures and corpus callosum, (Harbaugh and Wilson, 1982; Velasco et al., 2021), respectively. Later animal studies demonstrated that electrical stimulation of the thalamus at slow frequencies synchronized remote brain activity, while higher frequencies desynchronized neocortical rhythms (Jasper et al., 1955). Taken together, these experiments demonstrated that whether generalized epilepsy originates in the brainstem or higher brain structures, the thalamus plays a central role in modulating generalized epileptic activity (Ryvlin et al., 2021). Moreover, results from intracranial recordings of epileptic patients demonstrated a relationship between dynamic cortico-thalamic oscillations and seizure propagation and termination (Evangelista et al., 2015). Accordingly, any progress in the development of stimulation paradigms targeting thalamocortical circuits demands a greater understanding of how the thalamus regulates the balance between excitation and inhibition within an epileptic network (Yu et al., 2018).

Several nuclei have been suggested as targets for deep brain stimulation (DBS) due to their connectivity, anatomical location within the thalamus, and positive results in animal models. Preeminently, The Papez circuit connects the thalamus with the mesial temporal lobe, and incorporates additional limbic and neocortical structures into tracts that carry epileptic activity (Krishna et al., 2016; Elder et al., 2019). This pathway links hippocampal projections to the mammillary bodies via the crura and fornix, terminating in synapses at the anterior thalamic nuclei (ANT). The circuit is completed by radiations from the ANT arching through the cingulum, passing through the parahippocampus, and arriving back at the hippocampus (Zangiabadi et al., 2019; Perez-Malagon and Lopez-Gonzalez, 2021). Animal studies have implicated the Papez circuit in seizure propagation, (Perez-Malagon and Lopez-Gonzalez, 2021) and pathologic alterations to the Papez circuit are implicated in several epilepsy syndromes (Zangiabadi et al., 2019). Moreover, the efficacy of ANT stimulation in limbic epilepsy supports the prominent role played by the ANT as a conduit for epileptic discharges (Burdette et al., 2020). Given that many seizures propagate along the Papez and other cortical-striatal-thalamic circuits, an emerging goal of neuromodulation has been to interrupt the spread of seizures at deep brain nodes remote to epileptogenic cortex (Rincon et al., 2021). Previous reports of open-loop ANT stimulation have suggested that this modality is most effective in cases where there is no structural abnormality detected on brain imaging. Conversely, patients with dysplasia or cortical atrophy tend to demonstrate minimal benefit from ANT stimulation (Piacentino et al., 2015). Observations showing increased metabolic activity during seizures, as well as the effects of inhibitory stimulation or palliative lesions in the ANT in animal models of epilepsy highlight the role of these nuclei in facilitating seizure propagation (Mirski et al., 1997; Hodaie et al., 2002). Second, the centromedian thalamic nuclei (CMT) receives converging projections from many regions involved in seizure propagation, including the neocortex, basal ganglia and brainstem. The CMT is also active in regulating attention and arousal, and pathologic, low-frequency signaling in the CMT has been correlated with loss of consciousness during epilepsy (Welch et al., 2021). Previous studies have demonstrated that stimulation in the CMT is effective in a spectrum of generalized epilepsies- including absence seizures- in adults and children (Welch et al., 2021). Moreover, the relatively large size and convenient localization of the CMT bilateral to the third ventricle have made the CMT an attractive target for therapeutic DBS in other neurologic disorders including Tourette’s syndrome Parkinson’s disease (Ilyas et al., 2019). Third, the pulvinar nuclei (PVN) are the largest thalamic nuclei, and are heavily interconnected with posterior neocortical regions involved in visual processing and memory (Burdette et al., 2021). Their extensive connections to the posterior parieto-temporal lobes and the occipital lobes make these structures an attractive target for epilepsy arising in or near eloquent, posterior cortex (Burdette et al., 2021).

The efficacy of disrupting thalamic function in controlling seizures has been recognized for decades (Takebayashi et al., 2007). Monkey and cats with epileptogenic temporal lesions showed that destruction of the ANT significantly reduced seizure duration and frequency, (Kusske et al., 1972) providing early evidence that the ANT play a prominent role in the generalization of temporal epilepsy (Child and Benarroch, 2013). A rodent model of chemically induced generalized seizures demonstrated that injecting muscimol, a γ-aminobutyric acid (GABA)-agonist, into the ANT increases the seizure threshold and desynchronizes cortico-thalamic EEG activity (Mirski and Ferrendelli, 1986). More recently, chemogenetics were utilized in the reversible inhibition of midline and intralaminar thalamic nuclei to block seizures originating in the rodent amygdala (Wicker and Forcelli, 2016). In their review of ANT physiology and pathophysiology, Child and Benarroch implicate burst firing patterns of thalamic neurons, caused by aberrant T-type calcium channel currents, in propagating seizure activity (Child and Benarroch, 2013). Recordings of the ANT in awake patients with severe temporal epilepsy demonstrated bursting activity associated with low threshold spiking in thalamic neurons (Hodaie et al., 2006). The response of thalamic neurons to excitatory cortical input occurs as either phasic or tonic firing, depending on the activation or inactivation of voltage-dependent T-type calcium channels, which facilitate depolarization; this activity is not seen in rodent knockouts of T-type calcium channels, which are resistant to experimentally induced absence seizures (Kim et al., 2001; Sherman, 2001; Child and Benarroch, 2013). Burst firing of the thalamic neurons in the setting of generalized epilepsy represents the de-inactivation of these inward-depolarizing calcium channels when the neurons are hyperpolarized, leading to low-threshold calcium currents and a burst- firing pattern (Child and Benarroch, 2013). Attention and awareness are modulated by thalamo-cortical feedback loops, and loss of consciousness during absence seizures is believed to be caused by disruption of these circuits by propagation of pathologic 3-Hz spike and wave discharges. Accordingly, Kokkinos et al. (2020) posit that responsive stimulation of the CM after detection of these aberrant postsynaptic potentials desynchronizes the loops carrying epileptic, low-frequency rhythms, rescuing the endogenous, high-frequency rhythms and rescues the endogenous high frequency activity which maintains consciousness. Additionally, Herlopian et al. (2019) inferred from ECoG and scalp EEG that a localized, epileptic circuit existed between the ANT and cortical seizure focus which produced a generalized seizure phenotype in the absence of widespread epileptic discharges across the cortex. Disruption of this ANT-cortical loop was able to significantly reduce the frequency of absence seizures in their patient (Herlopian et al., 2019).

Although limited in quantity, published results of RNS in both the ANT and CMT for generalized epilepsy and absence seizures have been positive. The decision to target one or the other has thus far been operator-dependent, and further progress is needed to predict which target would be most efficacious in each patient (Herlopian et al., 2019). Nonetheless, the degree to which ANT stimulation recruits cortical rhythms has been used as a predictor of success for ANT DBS in patients with TRE (Hodaie et al., 2002).

As noted previously, animal models of epilepsy have shown that lesions or high frequency stimulation specifically in the ANT, or its afferent projections, reduce seizure frequency (Kusske et al., 1972; Hodaie et al., 2002). Consistent with this data, several investigators have noted immediate improvement in patients treated with ANT electrode implants, even prior to delivery of stimulation. Some have attributed the more immediate therapeutic benefits of thalamic DBS implants to a “microthalamotomy” effect, where the implant causes a transient functional lesion in the ANT and/or its associated epileptic circuitry (Tasker, 1998; Hodaie et al., 2002; Burdette et al., 2020). This phenomenon likely explains the positive results seen acutely (i.e., within days to weeks of beginning treatment) in the subjects treated with the externalized system described by Osorio et al. (2005). While common, this effect is not universal. In one report, 15/16 subjects undergoing open loop stimulation in the ANT for refractory epilepsy demonstrated persistent insertional effects on seizure frequency for at least 1 year following lead implantation, while these effects were not detected in the remaining patient (Krishna et al., 2016). In another case series, four subjects received bilateral ANT implants and no significant improvement in seizures was detected for any patient during the first 6 weeks of treatment with open loop stimulation (Osorio et al., 2007).

Thomas and Jobst (2015) suggest enhancement of GABA-mediated hyperpolarization may be responsible for the therapeutic action of responsive neurostimulation on aborting nascent seizures. They cite additional studies demonstrating that high frequency stimulation upregulates glutamate breakdown while downregulating the activity of calmodulin-dependent protein kinase II, resulting in inhibition of the stimulated neurons (Thomas and Jobst, 2015). Moreover, the results of the RNS Pivotal trial showed that the benefits of chronic ANT stimulation increase over time, supporting the notion that DBS at remote locations have a multitude of lasting effects on distant neurons with connections to the target. Proposed mechanisms include network rearrangement and effects on mRNA transcription and protein synthesis in neurons throughout the circuit (Osorio et al., 2005; Andrade et al., 2006; Thomas and Jobst, 2015).

Closed- and open-loop thalamic stimulation is being increasingly pursued in clinical settings to treat refractory epilepsy (Sisterson et al., 2019). Despite increasing empirical data supporting the safety and efficacy of this treatment modality, several knowledge gaps persist in the field. First, there is no standardized selection criteria for thalamic stimulation candidates. There is also limited information guiding when targeting the thalamus with open-loop stimulation vs. RNS is appropriate. While chronic DBS is supported by large trials and is approved for epilepsy, closed loop has fewer neurocognitive side effects and sleep disruption (Burdette et al., 2020, 2021; Kokkinos et al., 2020). RNS confers the additional advantage of isolating intracranial ictal and interictal recordings, allowing clinicians to keep track of how the seizure disorder reacts to stimulation (Burdette et al., 2020). Finally, closed-loop systems require less energy, which conserves battery life, resulting in fewer surgical procedures (Kwon et al., 2020). The question of whether responsive stimulation is more efficacious than continuous thalamic DBS remains to be addressed, as the two techniques have not been directly compared in the same subjects (Burdette et al., 2020). There currently are no biomarkers which would predict the efficacy of RNS system for any approved or off-label applications (Ryvlin and Jehi, 2022).

Clinicians have noted several challenges while employing the RNS system to target cortical and deep brain structures (Sisterson et al., 2019; Welch et al., 2021). Over months to years of surveillance, the system generates large datasets which need to be filtered and analyzed by the clinical team. The RNS system partially improves this temporal resolution by selectively tagging and storing sections of ECoG activity which occur before and after ictal events that trigger stimulation. Additionally, patients can perform a magnetic scalp swipe to activate ECoG recording at the onset of symptoms. The latter strategy ensures recording of epileptic episodes which do not trigger stimulation and identifies ECoG activity not previously implicated by the clinical team (Jarosiewicz and Morrell, 2021). Moreover, despite the customizability afforded by the programmable RNS system, there are as yet no systems which can titrate and optimize the stimulation paradigm in real time in response to ECoG surveillance, or even within a period that maximizes the effectiveness of neurostimulation (Sisterson et al., 2019; Ganti et al., 2022). The thalamic nuclei are not routinely implanted in diagnostic stereo-EEG, (Ganti et al., 2022) and while future studies utilizing deep learning algorithms may identify reliable seizure patterns, to date, there is not sufficient thalamic ECoG data to power such an undertaking (Ganti et al., 2022). Likewise, there is currently no way to evaluate the clinical efficacy of closed-loop detection and stimulation in response to individual seizure events, but rather the performance of RNS is evaluated by assessing the patient’s overall seizure frequency and severity over the entire follow-up period (Sisterson et al., 2019). Put differently, there currently is no way to calculate or improve the sensitivity of the RNS system for any given patient, where the detection and stimulation parameters are set based on a combination of manufacturer recommendations, results of previous studies, and the patient’s RNS records (Sisterson et al., 2019). Another obstacle is presented by insurance companies which often require multiple appeals and extensive advocacy by the surgical team on behalf of the patient, (Kokoszka et al., 2018) and which may pose a significant barrier at centers that do not have a history implanting RNS in off-label settings. Ideally, the results of these reports and future studies can serve as important precedent for patients and their providers seeking insurance authorization for thalamic RNS in treating TRE (Kokoszka et al., 2018).

While reports of off-label responsive thalamic stimulation in epilepsy are generally encouraging, the evidence supporting this modality has several limitations. First, the evidence is comprised of a small dataset of 29 subjects, of whom 5 were treated for 1 month or less. Second, these reports are themselves limited by small, unmatched cohorts. Third, the 29 subjects described herein received thalamic stimulation at an advanced phase of their illness, after having failed years of medication trials, many of whom had additionally undergone epilepsy surgeries and/or VNS implants. Whereas this stage of illness is characteristic of RNS candidates, it limits the generalizability and predictive value of these results in patients who may consider RNS as an earlier line of treatment for TRE (Thomas and Jobst, 2015). Accordingly, it is challenging to generalize the improvements seen in these severely affected individuals to the wider epilepsy population. In addition, five of the subjects had complete or partial callosotomy, while nine subjects underwent some form of cortical resection and one report described five subjects with previous “unspecified” epilepsy surgeries (Burdette et al., 2020). It is difficult to predict how these changes in neuroanatomy impacted the efficacy of thalamic RNS, and, by extension, it is difficult to make inferences from these results to the general epilepsy population (Herlopian et al., 2019). It is also challenging to make deductions from these studies regarding the effects of thalamic responsive stimulation on neurocognitive wellbeing, as only 1 out of 10 publications included formal neurocognitive testing at follow-up (Burdette et al., 2021). Furthermore, whereas larger studies utilize responsive stimulation for several years before assessing the impact on seizure frequency, (Ryvlin and Jehi, 2022) follow-up data for 12/29 of the subjects described in this review was obtained within 12 months from starting thalamic stimulation (Osorio et al., 2005; Kokoszka et al., 2018; Burdette et al., 2020, 2021; Kwon et al., 2020; Welch et al., 2021; Beaudreault et al., 2022). Finally, although seizure frequency was a primary outcome in all 10 studies, the RNS system cannot record for extended periods of time and does not track all data relating to seizure frequency (Thomas and Jobst, 2015). Consequently, the authors’ reliance on self-reports for tracking seizure outcome inevitably introduces some degree of recall bias (Elder et al., 2019).

## Conclusion

The data supporting off-label closed-loop thalamic stimulation is limited to 29 adult and pediatric patients with TRE, many of whom experienced significant improvement in seizure duration and frequency. This encouraging progress must be verified in larger studies.

## Author Contributions

CF: conceptualization, methodology, investigation, data curation, writing–original draft, and project administration. EE: conceptualization, writing–review and editing, and supervision. All authors contributed to the article and approved the submitted version.

## Conflict of Interest

The authors declare that the research was conducted in the absence of any commercial or financial relationships that could be construed as a potential conflict of interest.

## Publisher’s Note

All claims expressed in this article are solely those of the authors and do not necessarily represent those of their affiliated organizations, or those of the publisher, the editors and the reviewers. Any product that may be evaluated in this article, or claim that may be made by its manufacturer, is not guaranteed or endorsed by the publisher.

## Footnotes

1 Available online at: https://www.epilepsy.com/release/2014/3/fda-approvesresponsive-neurostimulation-therapy-neuropace (accessed May 17, 2021).
